# Tick Salivary Kunitz-Type Inhibitors: Targeting Host Hemostasis and Immunity to Mediate Successful Blood Feeding

**DOI:** 10.3390/ijms24021556

**Published:** 2023-01-13

**Authors:** Mohamed Amine Jmel, Hanne Voet, Ricardo N. Araújo, Lucas Tirloni, Anderson Sá-Nunes, Michail Kotsyfakis

**Affiliations:** 1 Laboratory of Genomics and Proteomics of Disease Vectors, Institute of Parasitology, Biology Centre, Czech Academy of Sciences, 37005 Ceske Budejovice, Czech Republic; 2 Laboratory of Hematophagous Arthropods, Department of Parasitology, Federal University of Minas Gerais, Belo Horizonte 31270-901, MG, Brazil; 3 National Institute of Science and Technology in Molecular Entomology, National Council for Scientific and Technological Development (INCT-EM/CNPq), Rio de Janeiro 21941-902, RJ, Brazil; 4 Tick-Pathogen Transmission Unit, Laboratory of Bacteriology, Rocky Mountain Laboratories, National Institute of Allergy and Infectious Diseases, National Institutes of Health, Hamilton, MT 59840, USA; 5 Laboratory of Experimental Immunology, Department of Immunology, Institute of Biomedical Sciences, University of Sao Paulo, Sao Paulo 05508-000, SP, Brazil

**Keywords:** Kunitz-type, protease inhibitors, ticks, hemostasis, immunomodulation, parasite-host interactions

## Abstract

Kunitz domain-containing proteins are ubiquitous serine protease inhibitors with promising therapeutic potential. They target key proteases involved in major cellular processes such as inflammation or hemostasis through competitive inhibition in a substrate-like manner. Protease inhibitors from the Kunitz superfamily have a low molecular weight (18–24 kDa) and are characterized by the presence of one or more Kunitz motifs consisting of α-helices and antiparallel β-sheets stabilized by three disulfide bonds. Kunitz-type inhibitors are an important fraction of the protease inhibitors found in tick saliva. Their roles in inhibiting and/or suppressing host homeostatic responses continue to be shown to be additive or synergistic with other protease inhibitors such as cystatins or serpins, ultimately mediating successful blood feeding for the tick. In this review, we discuss the biochemical features of tick salivary Kunitz-type protease inhibitors. We focus on their various effects on host hemostasis and immunity at the molecular and cellular level and their potential therapeutic applications. In doing so, we highlight that their pharmacological properties can be exploited for the development of novel therapies and vaccines.

## 1. Introduction

Many arthropods developed hematophagy over an evolutionary timescale of millions of years with the primary objective of feeding on vertebrate blood [1]. This evolution included the development of complex physiological and molecular mechanisms to circumvent vertebrate host defense mechanisms, such as hemostasis and immunity [2]. These mechanisms developed from the cretaceous era onwards across over 500 arthropod genera comprising at least 19,000 species [3]. Of these arthropods, ticks have received particular attention due to their worldwide distribution and veterinary and medical importance [4]. Ticks are the second most common vector of pathogens after mosquitos and cause important human diseases including tick-borne encephalitis and Lyme borreliosis [5].

Ticks have therefore evolved and developed unique strategies to escape vertebrate host defenses to remain attached and complete a long-lasting blood meal [6,7]. To achieve this, ticks release their saliva—a complex mixture of pharmacologically bioactive compounds (peptides/proteins, lipids, nucleic acids, and other molecules) [8]—at the bite site to avoid host defenses such as hemostasis, inflammation, and innate and adaptive immunity [9]. To maintain host blood fluidity, tick saliva contains specific proteins and low molecular weight molecules that specifically target hemostatic cascades and consequently block the triad of blood coagulation, platelet aggregation, and vasoconstriction [10]. These antihemostatic molecules have been extensively investigated, as their manipulation may prevent ticks from feeding and, consequently, pathogen transmission [11]. Furthermore, recent advances in high-throughput technologies (transcriptomics, proteomics, among others) have rapidly expanded our understanding and identification of tick salivary gland and midgut proteins that act at the tick–host interface to facilitate pathogen transmission [12].

Several proteomic and transcriptomic studies of the saliva and salivary glands (to produce sialoproteomes and sialotranscriptomes, respectively) of infected and non-infected ticks have revealed differences in the expression profiles in the presence or absence of pathogens [13]. As host hemostasis and immune responses are tightly regulated by proteases, tick saliva is particularly rich in protease inhibitors (PIs), of which only a small fraction has been biochemically and functionally characterized [8,9]. PIs regulate several biochemical processes to prevent protease activity that might harm the host organism [14]. At the tick–host interface, these PIs act in favor of the ectoparasite by neutralizing host proteases involved in immune responses and hemostasis [15]. Depending on their targets, tick salivary PIs can be subclassified such as serine, cysteine, aspartyl, threonine, and glutamate proteases [5]. Since the serine proteases are the most abundant protease family [16], their respective inhibitors represent the largest group of PIs in animals, plants, and microorganisms and are themselves divided into superfamilies including Kunitz-type protease inhibitors, Bowman–Birk inhibitors, serpins, Kazal-type protease inhibitors, thryropin, and trypsin inhibitor-like domain (TIL) inhibitors [11].

Members of the Kunitz-type inhibitor superfamily are among the most abundant PIs in tick saliva and, in most cases, they inhibit trypsin-like serine proteases [17]. Members of this superfamily contain at least one characteristic Kunitz domain: a 3D structure formed from α-helices and anti-parallel β-sheets compacted by three disulfide bonds [18]. Kunitz-type inhibitors can contain several Kunitz motif repeats, and proteins with a single (monolaris), double (bilaris), or up to five (penthalaris) motifs have been reported in tick saliva [19].

In this review, we provide an overview of current knowledge about Kunitz-type inhibitors, their structure, and their inhibitory mechanisms. We also describe the various reported effects of tick salivary Kunitz-type inhibitors on host hemostasis and immunity to provide insights into their potential use as immunobiologics and vaccines against ticks and tick-borne diseases.

## 2. Kunitz-Type Inhibitors: Low Molecular Weight Serine Protease Inhibitors

Kunitz domain-containing proteins are serine PIs found in almost all living organisms including animals, plants, and microbes. The first identified member of this family, bovine pancreatic trypsin inhibitor (BPTI), was described over 80 years ago [20] and is one of the most extensively studied globular proteins due to its use as a model system for protein structure and folding investigations [21]. In addition to their serine PI activity, some Kunitz domain-containing proteins can act as ion channel blockers [22], especially in the venom of poisonous animals, so are also called Kunitz-type toxins, although they have also been reported in parasite secretions [22,23]. While Kunitz-type PIs are essential regulators of inflammatory processes in vertebrates, their function in invertebrates is broad, with anticoagulant, fibrinolytic, and antimicrobial activities all described [17]. Structurally, these proteins are typically small molecules with a molecular weight between 18 and 24 kDa [24]. A Kunitz domain is usually 60 amino acid long, weighs around 7 kDa, and consists of two antiparallel β-strands and one or two α-helices [24]. The Kunitz domain contains six conserved cysteines forming three disulfide bridges that stabilize the structure, one of them stabilizing the two binding domains [25].

Most Kunitz-type inhibitors are competitive inhibitors that bind reversibly to the active site in a substrate-like manner [26]. The protease-binding loop formed by the Kunitz-type domain executes protease inhibition by tightly but non-covalently binding the serine protease active site. The most exposed region of the loop harbors position P1, which is the reactive site and the critical determinant of inhibitor recognition specificity [17,25]. The enzyme is thus blocked without any conformational changes, and the Kunitz inhibitor forms an anti-parallel β-sheet between the enzyme and inhibitor, where numerous non-covalent interactions ensure tight binding [17]. The reactive center loop (RCL) of Kunitz-type inhibitors is structurally adapted to a wide panel of proteases, which explains why several tick salivary Kunitz-type inhibitors have multiple targets [27]. Conversely, some tick-derived Kunitz-type inhibitors targeting thrombin do not follow the canonical mechanism of protease inhibition. Instead, their N-terminal residues bind across the thrombin active site cleft, while their C-terminal modules interact with the basic exosite I of the protease [1]. The pluripotency of these PIs and their implication in various pathways including hemostasis, inflammation, immunomodulation, or tumor biology make them very attractive candidates as therapeutics and also establishes them as valuable tools for biochemical studies.

## 3. Tick Saliva as a Source of Bioactive Kunitz-Type Inhibitors

As noted above, serine PIs are abundant in tick salivary glands, where they play a role in blocking vertebrate host responses to guarantee success of blood feeding. Of the four main classes of serine PIs, proteins with Kunitz domains are usually the most represented in salivary gland transcriptomes, both in terms of number and expression levels [28]. This abundance is seen in both the argasid (soft tick) and ixodid (hard tick) families at all development instars (larvae, nymphs, and adults), although there is marked variability in different tick species [29,30].

In tick salivary gland transcriptomes, Kunitz-type domains are usually found within the five most expressed classes of protein along with lipocalins, basic and acid tail proteins, and proteases. Their relative expression levels vary between tick species, and in argasids of the genus *Ornithodoros* (e.g., *O. brasiliensis*, *O. erraticus*, and *O. moubata*) and some ixodid species (e.g., *Amblyomma triste*), Kunitz proteins are the fourth or fifth most abundant class [30,31,32,33]. For some tick species of the *Amblyomma* genus, proteins with Kunitz domains are even more abundant, being the first or second most commonly expressed, as seen for *A. tuberculatum*, *A. americanum*, *A. sculptum* (sin *A. cajennense*), and *A. parvum* [33,34,35]. Additionally, a dataset of high-quality expressed sequence tags (ESTs) from the eight libraries of *Rhipicephalus (Boophilus) microplus* (RMallHxN) estimated that up to 1% of the total putative secreted proteins of the species are from the Kunitz-type inhibitor superfamily [36].

In general, Kunitz-type inhibitors account for 1 to 10% of reads of secreted proteins in salivary gland transcriptomes. However, their expression levels increase over the course of feeding over an estimated three-fold range [30]. This increase in expression varies according to each protein, with some showing no regulation during feeding and others showing substantial upregulation (Table 1); for example, AsKunitz transcripts are ~18.8 million-fold upregulated one day after the start of feeding [29].

The number of contig IDs of Kunitz-type proteins is also highly variable between tick species, from as low as 24–42 proteins in some transcriptomes (as seen for the argasids *O. rostratus* and *O. brasiliensis* [30,37] and the ixodid *A. tuberculatum* [34]) to over 100 in *O. moubata* [32] and *O. erraticus* [31]. An extensive database of tick salivary proteins from forty-four species from ten genera showed that six classes contain the majority of sequences, and Kunitz-type PIs represent the second most abundant class with 1882 proteins [28]. The function of most of these proteins is still unknown, although a few have been functionally characterized.

## 4. Hemostasis Modulation by Tick Salivary Kunitz Inhibitors

Following the cutaneous and vascular injury caused by the introduction of the tick mouthparts into the host, the vertebrate host initiates different but interrelated mechanisms including inflammation and other immune reactions triggered by different endogenous and/or exogenous factors [2,10]. Here we focus on the effect of tick salivary Kunitz-type inhibitors on hemostasis and host immune responses, although it should be noted that the effects of these protease inhibitors are wider [1].

With the primary objective of preventing blood loss, hemostasis encompasses three major synchronized mechanisms to achieve that objective [2]: vasoconstriction, blood coagulation, and thrombus formation/platelet aggregation. Vasoconstriction occurs after the release of several compounds such as leukotrienes by mast cells, endothelins by the local endothelium, serotonin and thromboxane by activated platelets, and other blood proteins such as angiotensin I [2,38]. The coagulation cascade represents an interconnected network of enzymatic cascades with several amplification and regulatory mechanisms [39]. With its extrinsic and intrinsic pathways, blood coagulation produces the fibrin needed for platelets to form the thrombi [40]. Consequently, platelets are another key actor in thrombus formation that assemble through fibrin binding to their αIIbβ3 integrin [2].

Despite the complexity of host hemostatic responses, ticks have developed several evasive measures to block hemostasis, since maintaining availability of vertebrate host blood is essential to survival [10,25]. As noted above, Kunitz-domain PIs are highly represented in tick saliva and constitute the largest group of serine PIs [2]. In 1990, the first tick anticoagulant peptide (TAP) belonging to the Kunitz superfamily was purified from a whole-body extract of *O. moubata* and showed high specificity for factor X (FX) [41]. TAP displayed interesting anti-hemostatic effects, including inhibiting thromboplastin-induced fibrinopeptide production in monkeys or significantly inhibiting thrombosis in an arterial thrombosis model in its recombinant form (rTAP) [41,42]. Thus, TAP stimulated interest in tick salivary glands as a source of Kunitz-type inhibitors with important anti-hemostatic properties and therefore potential therapeutic value. Following on from studies of TAP, disagregin was the first inhibitor of platelet aggregation and platelet adhesion to fibrinogen directly isolated from tick salivary gland extracts (also from *O. moubata*) [43]. Since then, many other Kunitz-type inhibitors have been reported in hard ticks that target the coagulation cascade and platelet aggregation through high potency for key serine proteases such as trypsin, elastase, thrombin, or FX [2]. For instance, Ir-CPI isolated from the salivary glands of *Ixodes ricinus* was reported as a multifunctional Kunitz-type inhibitor that reduced venous thrombus formation in rat and mouse venous and arterial thrombosis models. Moreover, Ir-CPI protected against thromboembolism induced by epinephrine or collagen [44].

Other Kunitz-type inhibitors have since shown anti-angiogenic and anti-tumor activities. Ixolaris from *Ixodes scapularis* [18] and Amblyomin-X from *A. sculptum* (a member of *A. cajennense* species complex) [39] showed potent anti-hemostatic activities, mainly related to FX inhibition. Amblyomin-X was reported as a non-competitive inhibitor of FX with a consequent inhibitory effect on prothrombinase and tenase complexes [39,45]. Conversely, Ixolaris inhibits FX through exosite binding to form a complex with factor VIIa (fVIIa) and tissue factor (TF) [46]. Moreover, by inhibiting the extrinsic pathway, Ixolaris showed concentration-dependent inhibition of thrombus formation in a venous thrombosis model [47].

Table 1 provides an up-to-date list of salivary Kunitz-type inhibitors originating from ticks with different hemostasis-related properties. In addition to anti-tumor and anti-angiogenic activities, other tick salivary Kunitz-type inhibitors with immunomodulatory activities have been reported, as discussed below.

**Table 1:** Kunitz-type inhibitors characterized in tick saliva.

Kunitz Protein	Tick Species	Number of Kunitz Domains	Target Protease(s)	Biological Effect	Transcriptomic Induction/Elevation by Blood Feeding	Vaccine-Related Study (Observed Effect on Ticks)	Reference
**HA11**	*Hyalomma asiaticum*	Monolaris		Anticoagulant (intrinsic pathway)	Yes	Yes (reduced engorged body weight)	[48]
**Rhipilin-1**	*Rhipicephalus hemaphysaloides*	Monolaris		Anticoagulant (intrinsic pathway)	Yes	No	[49]
**Rhipilin-2**	*Rhipicephalus hemaphysaloides*	Monolaris	Trypsin, elastase	Anticoagulant (intrinsic pathway)	Yes	No	[50]
**AsKunitz**	*Amblyomma sculptum*	Monolaris	Thrombin	Anticoagulant, anti-complement (classical and alternative pathways)	Yes	Yes (reduced egg hatching, increased mortality)	[29]
**Amblyomin-X**	*Amblyomma sculptum*	Monolaris	FXa	Anticoagulant, antithrombotic, antiangiogenic, antitumor (reduces tumor growth and metastasis, induces apoptosis in tumor cell lines)	No	No	[39]
**Amblin**	*Amblyomma hebraeum*	Bilaris	Thrombin	Anticoagulant	No	No	[51]
**IrSPI**	*Ixodes ricinus*	Monolaris	Elastase	Immunomodulatory (repression of proliferation of CD4^+^ T lymphocytes and proinflammatory cytokine secretion from both splenocytes and macrophages)	Yes	Yes (increased engorgement, decreased mortality, increased molting)	[52,53]
**Ir-CPI**	*Ixodes ricinus*	Monolaris	FXIa, FXIIa, kallikrein	Anticoagulant (intrinsic pathway), antifibrinolytic, antithrombotic	No	No	[44]
**Ixolaris**	*Ixodes scapularis*	Bilaris	FX(a)	Anticoagulant (extrinsic pathway), antithrombotic, antiangiogenic, antitumor	No	No	[47,54]
**Penthalaris**	*Ixodes scapularis*	Pentalaris	Fx(a)	Anticoagulant	No	No	[55]
**Tryptogalinin**	*Ixodes scapularis*	Monolaris	Human skin β-tryptase, matriptase, plasmin, elastase, α-chymotrypsin, trypsin	Not characterized yet	No	No	[56]
**Ra-KLP**	*Rhipicephalus appendiculatus*	Monolaris	No anti-protease activity	Activates maxiK channels	Yes	No	[57]
**Boophilin**	*Rhipicephalus microplus*	Bilaris	Thrombin, trypsin, plasmin, FXIa, kallikrein, elastase	Anticoagulant, platelet antiaggregant	No	No	[58,59]
**rBmTI-A**	*Rhipicephalus microplus*	Bilaris	Trypsin, kallikrein, elastase, plasmin	Anti-inflammatory, antiangiogenic; protective role in pulmonary disorders (emphysema and allergic inflammation)	No	No	[60,61,62,63,64]
**rBmTI-6**	*Rhipicephalus microplus*	Trilaris	Trypsin, plasmin	Attenuates inflammation in elastase-induced emphysema	No	No	[65,66]
**Haemangin**	*Haemaphysalis longicornis*	Monolaris	Trypsin, chymotrypsin, plasmin	Anti-angiogenic (inhibits proliferation and induces apoptosis of endothelial cells), modulates wound healing	Yes	No	[67]
**HlMKI**	*Haemaphysalis longicornis*	Monolaris	*Haemaphysalis longicornis* trypsin-like serine proteinase (HlSP)	Not characterized yet	Yes	No	[68]
**HlChI**	*Haemaphysalis longicornis*	Monolaris	Chymotrypsin, trypsin	Not characterized yet	Yes	No	[69]
**KPI**	*Dermacentor variabilis*	Pentalaris	Trypsin	Anticoagulant (intrinsic pathway)	Yes (in midgut)	No	[70]
**Ornithodorin**	*Ornithodoros moubata*	Bilaris	Thrombin	Not characterized yet	No	No	[71]
**Disagregin**	*Ornithodoros moubata*		No anti-protease activity	Platelet antiaggregant	No	No	[72]
**Savignygrin**	*Ornithodoros savignyi*	Monolaris	No anti-protease activity	Platelet antiaggregant	No	No	[73]

## 5. Modulation of Host Inflammation and Immunity by Tick Salivary Kunitz Inhibitors

Although a Kunitz-type inhibitor in ticks was first described in 1990, study of their role in tick–host relationships was largely restricted to hemostasis over the following 20 years. The first evidence of their potential activity in tick immunity came from two related studies demonstrating that *Dermacentor variabilis* expresses a Kunitz-type inhibitor in its midgut capable of limiting *Rickettsia montanensis* growth/invasion both in vitro [70] and in vivo [74]. Such control of bacterial growth by a Kunitz-type inhibitor, observed in nodules of winged bean plants colonized by *Rhizobium* spp. [75], has opened up new avenues for evaluating the role of Kunitz-type inhibitors in host inflammation and immunity. However, compared with other protease inhibitors (e.g., serpins and cystatins), the activities of salivary Kunitz-type PIs in vertebrate immune phenotypes are far less studied and most remain elusive.

**Figure 1:**
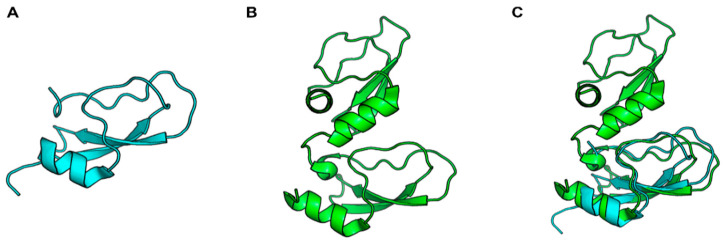
Cartoon representation of the second Kunitz domain of human TFPI (PDB: 1TFX) (**A**), the two Kunitz domains of Ixolaris (PDB: 6NAN) (**B**), and the superposition of the two mentioned Kunitz domains (**C**).

The bidirectional interface between coagulation and inflammation is now well established [76]. For example, TF is the main activator of clotting under physiological conditions, and several studies have now demonstrated a TF-dependent coagulation–inflammation circuit [77]. The sialotranscriptome of *I. scapularis* revealed a sequence with homology to TF pathway inhibitor (TFPI) with two Kunitz-like domains. The expressed recombinant protein was named Ixolaris (Figure 1) and characterized as an inhibitor of FVIIa/TF-induced FX activation in the picomolar range [18]. Interestingly, TF-expressing monocytes produce more proinflammatory cytokines IL-1β, IL-6, and TNF-α than TF-negative monocytes when stimulated by LPS. However, although Ixolaris inhibited TF function in LPS-stimulated monocytes *in vitro*, it did not affect TF expression or proinflammatory cytokine production in these cells [78]. Penthalaris, another TFPI homolog found in the *I. scapularis* sialotranscriptome containing five tandem Kunitz domains, also inhibits FX activation through the FVIIa/TF pathway. However, its potential role in inflammation still needs to be established [55].

*Rhipicephalus* spp. have atypical Kunitz/BPTI proteins in their saliva that target unusual proteases. For example, *Rhipicephalus sanguineus* TdPI (from Tick-derived Protease Inhibitor) is a salivary protein with a modified Kunitz fold and disulfide-bond pattern that inhibits human β-tryptase, a human mast cell-derived serine protease [79]. Interestingly, when injected into mouse ears, TdPI accumulated in the cytoplasmic granules of dermal mast cells and was detectable for two days [79]. Given the role of mast cell tryptase in inflammation and allergy [80], it can be hypothesized that TdPI treatment might work as a mast cell-stabilizing agent, either suppressing the release of mast cell-derived mediators or neutralizing tryptase release, with a clear benefit for tick feeding. Of note, *I. scapularis* also possesses a Kunitz sequence closely related to TdPI that displays an unusual cysteine motif compared with other Kunitz-type inhibitors; this inhibitor was named tryptogalinin and it also targets human β-tryptase, suggesting similar functions to TdPI [56]. On the other hand, *R. appendiculatus* secretes a Kunitz/BPTI-like protein (*Ra*-KLP) with an extensive modification in its Kunitz fold and devoid of any anti-protease or anti-hemostatic activity. However, it does have a stimulatory effect on large-conductance Ca^2+^-activated K^+^ (maxiK) channels [57], similar to the Kunitz-type toxins described in helminths and poisonous animals [22,23]. Given these data, a machine learning algorithm was developed and validated in *I. ricinus* sialotranscriptomes to improve the identification Kunitz-domain proteins that also lack the PI function but interact with ion channels [81]. Of their many functions, this type of non-PI Kunitz proteins may act on elements of the cutaneous immune system, although such putative activities still require experimental proof.

Elastase contributes to many activities reported for neutrophils [82], and neutrophil elastase inhibitors are under investigation to treat a number of inflammatory conditions [83]. As a proof of concept, a recombinant preparation of *B. microplus* Trypsin Inhibitor A (rBmTI-A)—originally extracted from tick larvae—is a strong neutrophil elastase inhibitor and presented anti-inflammatory properties in experimental models of elastase-induced emphysema and other pulmonary inflammatory disorders [84]. Interestingly, both Rhipilin-2 from *R. hemaphysaloides* [50] and IrSPI from *I. ricinus* [52] inhibit elastase but no other enzymes typically targeted by Kunitz-type inhibitors. Thus, Rhipilin-2 and IrSPI are likely to be promising modulators of neutrophil function and neutrophil-associated inflammation. In addition, IrSPI has already been demonstrated to decrease CD4^+^ T cell proliferation and proinflammatory cytokine secretion by splenocytes and macrophages [52].

Some tick salivary Kunitz-type inhibitors display interesting effects on cell death. Haemangin, originally described in *Haemaphysalis longicornis* salivary glands, suppressed angiogenesis and would healing by inhibiting vascular endothelial cell proliferation and inducing apoptosis [67]. A transcript found in the sialotranscriptome of *A. sculptum*, coding a protein containing an N-terminal Kunitz-type domain and a C-terminus with no homology to any annotated sequences, was also identified and, like many other Kunitz-type proteins, it was initially shown to be an activated FX (FXa) inhibitor and to affect blood clotting in vitro and in vivo [85]. However, electrostatic potential mapping of its Kunitz-type region revealed a different pattern of charged residues compared with human TFPI-1 and TFPI-2, suggesting additional functional and structural features [86]. The molecule, named Amblyomin-X, in fact inhibited angiogenesis induced by VEGF-A (vascular endothelial growth factor A) in murine subcutaneous tissue and chicken chorioallantoic membrane models, delayed cell cycle progression, decreased cell proliferation and adhesion, and reduced tube formation and membrane expression of PECAM-1 (adhesion molecule platelet–endothelial cell adhesion molecule-1) [87]. In addition, Amblyomin-X showed antitumoral activity in vivo and in vitro, with investigation of its mechanisms of action revealing alterations in the ubiquitin-proteasome system and apoptosis induction [88]. Strikingly, these cytotoxic effects were selective to tumor cell lines (e.g., human melanoma, pancreatic adenocarcinoma, renal cell carcinoma) but not primary cells (human fibroblasts) nor non-tumor-derived cell lines, which may indicate that Amblyomin-X may not have off-target effects as a therapeutic drug [89,90,91].

In addition to Amblyomin-X, a second Kunitz-type inhibitor was identified in one of the *A. sculptum* sialotranscriptomes as the most expressed transcript of this family during blood feeding [33]. The recombinant protein, named AsKunitz, possesses eight cysteines, a typical Kunitz/BPTI domain, and inhibits thrombin but not FXa or trypsin. Among many activities, AsKunitz was the first salivary Kunitz shown to inhibit both the classical and alternative pathways of complement activation [29]. The complement system is considered an important line of defense against ticks since some of its components trigger mast cell degranulation and induce leukocyte recruitment to the skin [92], in addition to causing direct cell damage through the activation of the membrane attack complex [10]. This selective pressure by the host explains the high number anti-complement bioactive molecules found in tick saliva and midgut [93]. In addition, the complement system represents an important host effector mechanism against the pathogens transmitted by ticks [94,95]. Thus, by inhibiting the complement system components present in the blood, ticks assure a double-edged sword effect: the acquisition of a less harmful blood meal while enhancing the transmission of tick-borne diseases.

Table 1 also provides details of tick salivary Kunitz-type inhibitors with confirmed impacts on immunity in vertebrate hosts.

## 6. Kunitz-Type Inhibitors Used as Vaccine Antigens against Ticks and Tick-Borne Diseases

Anti-tick vaccines became commercially available in the early 1990s for the control of cattle tick infestations; they were the first commercial vaccines to target a multicellular ectoparasite [96]. More recently, evidence has accumulated that targeting tick proteins by vaccination not only reduces tick feeding and reproduction but also interferes with pathogen infection and transmission from the tick to the vertebrate host. However, despite the diversity of biochemical targets and functions in host immunity, only a few Kunitz-type inhibitors have been tested as antigen candidates in vaccination trials. The first was BmTI, purified from *R. microplus* larvae extracts, which elicited a protective immune response in vaccinated cattle with 72.8% efficacy and a 69.7% reduction in the number of adult females completing the parasitic phase of the life cycle [97]. However, when a synthetic peptide designed from BmTI N-terminal fragment was used in similar conditions, it offered only 18.4% protection against tick infestation in cattle [98]. Immunization of cattle with another trypsin inhibitor from *R. microplus* larvae (rRmLT), which resembles the three-headed Kunitz-type inhibitor BmTI-6, showed 32% efficacy against cattle tick infestation [99]. Only recently, a Kunitz-type inhibitor from tick saliva was tested as a tick vaccine. Mice immunized with AsKunitz, the anticoagulant inhibitor that also affects complement activation, showed >85% efficacy against challenge with adult female *A. sculptum*, while the mortality of nymphs fed on immunized mice reached 70% [29].

Besides immunization experiments, RNA interference (RNAi) has been shown to be a valuable tool for the study of tick gene function, characterization of the tick–pathogen interface, and screening and characterization of protective tick antigens [100]. RNAi is performed by inoculating double-stranded RNA (dsRNA) homologs of specific messenger RNAs (mRNA). This results in sequence-specific degradation, interference with gene expression, and subsequent loss of gene function. The Kunitz-type inhibitor hemalin is a thrombin inhibitor present in the midgut, salivary glands, hemocytes, and fat body of adult females and the nymphs and larvae of *H. longicornis* ticks. Silencing hemalin by RNAi led to a two-day extension of the tick blood feeding period, and 27.7% of RNA-treated ticks did not successfully complete blood feeding [101]. Haemangin was the second Kunitz-type inhibitor from *H. longicornis* ticks to have its biological function assessed by RNAi. Haemangin inhibits trypsin, chymotrypsin, and plasmin and disrupts angiogenesis and wound healing via inhibition of vascular endothelial cell proliferation and induction of apoptosis. Haemangin transcript is upregulated prior to complete feeding. Notably, RNAi-treated adult ticks had significantly diminished engorgement, while knock-down ticks failed to impair angiogenesis in vivo [67].

Boophilin, an anticoagulant and antithrombotic inhibitor of two Kunitz-type domains expressed in the intestine of fully engorged *R. microplus* females, inhibits thrombin, elastase, kallikrein, cathepsin G, and plasmin [58,59,102]. Silencing the boophilin gene by RNAi decreased egg production, indicating that boophilin expression is important but not vital, possibly due to functional overlap with other serine peptidase inhibitors in the *R. microplus* midgut. However, it is important to note that the RNAi experiment was performed using fully engorged females, and the role of boophilin during blood feeding was not evaluated [58]. Rhipilin-1 from *R. haemaphysaloides* ticks, homologous to TFPI, appears to be crucial for tick feeding, since it is transcribed in fed but not unfed ticks. Silencing Rhipilin-1 by RNAi led to a decreased rate of attachment and engorgement [49].

Given the level of structural and functional similarity between some vertebrate- and tick-derived Kunitz-type inhibitors, the development of vaccines against ticks using these proteins should include the evaluation of potential cross-reactivity of the antibodies generated. Although not likely and not reported in the scientific literature on the tick saliva-based vaccines, such cross-reactivity could possibly trigger some level of autoimmunity in the host.

## 7. Future Perspectives

**Figure 2:**
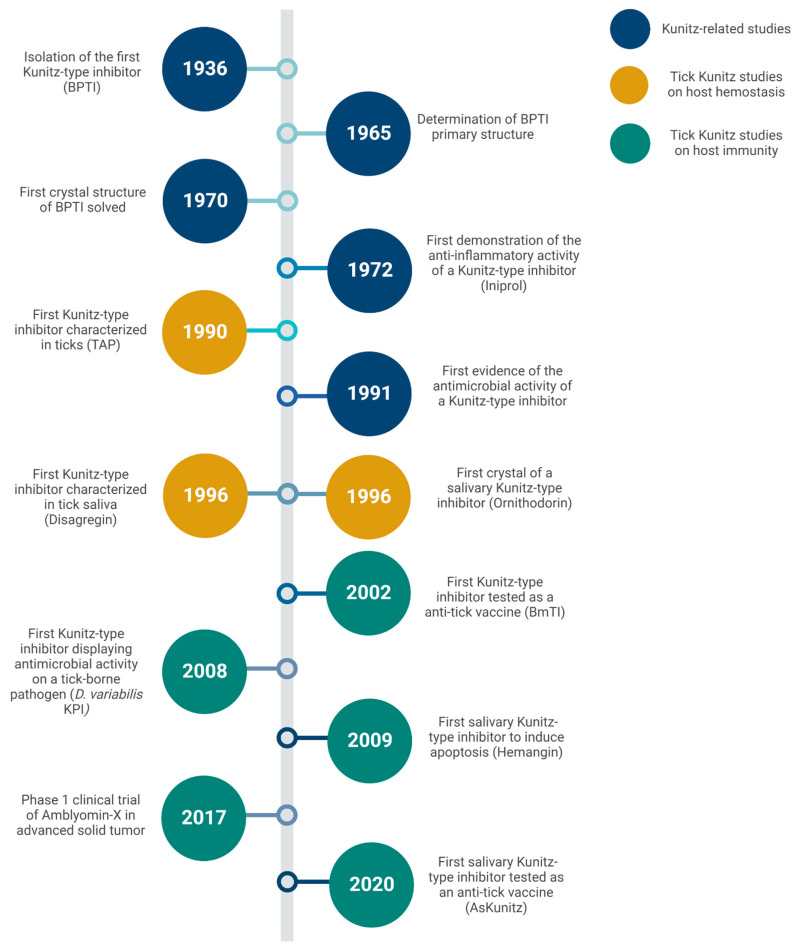
Discoveries and advances in Kunitz-type inhibitors since the uncovering of the family member and the major milestones in tick-derived species.

Kunitz-type inhibitors became part of the vocabulary of tick research about 30 years ago [41]. A timeline of discoveries and advances in this exciting area is depicted in Figure 2. During this time, there have been significant advances in the quantitative and qualitative technologies used to isolate, identify, and characterize salivary PIs (as for other biological active molecules derived from ticks). The fractionation of crude preparations (e.g., saliva or salivary glands extracts) followed by identification of individual molecules employed in earlier studies have been replaced with large-scale databases generated by high-throughput sequencing efforts of proteomes, transcriptomes, and, sometimes, genomes of different tick species. Since the pioneering sialome study that introduced a set of mRNAs and proteins expressed in the salivary glands of *I. scapularis* [101], dozens of similar studies have since been performed on several tick species, some of them revealing over a thousand potentially secreted proteins for each species. Initiatives such as TickSialoFam have been helping researchers to cope with the huge amount of information generated by these large-scale studies [28].

Among the seven categories of salivary proteins organized in TickSialoFam, PIs are the third largest in terms of relative number of sequences, and Kunitz-type inhibitors are by far the most abundant in this category, representing almost 70% of predicted proteins [28]. Such abundance contrasts with the 85 articles retrieved from PubMed using the search terms “ticks AND Kunitz” (as of November 2022) compared with 71 for “ticks AND serpin” and 54 for “ticks AND cystatin”, despite these families representing ~7% and ~4% of the predicted proteins, respectively. Even considering studies missing from the search or the strategy used, these results reinforce that research on tick salivary Kunitz-type inhibitors is still in its infancy.

Most early studies on Kunitz-type inhibitors from tick saliva focused on their anti-hemostatic activities. In fact, most of the inhibitors in this superfamily target proteases involved in coagulation. However, deeper biochemical characterization revealed unexpected new targets for some of the inhibitors, suggesting novel potential activities of Kunitz-type inhibitors in host immunity. While some of these activities still need to be experimentally confirmed, functional studies using synthetic or recombinant molecules have highlighted that Kunitz-type inhibitors are exciting anti-inflammatory, immunomodulatory, and antitumoral agents or vaccine antigens capable of blocking or decreasing tick infestation.

We believe that the next significant advance in the field will be the development of Kunitz-derived immunobiologics to treat clinical conditions and saliva-based vaccines against ticks or tick-borne diseases. Preparations containing these molecules have been patented around the world. At least one Kunitz-type inhibitor is already under investigation for human use after preclinical animal studies [102]. Amblyomin-X was approved for a Phase 1 clinical trial for patients with advanced solid tumors (https://clinicaltrials.gov/ct2/show/NCT03120130, accessed on 10 December 2022). In addition, some Kunitz-type inhibitors also showed anti-tick activity and protected against tick-borne diseases when used as a vaccine preparation either in experimental models or in real-world evaluations with natural tick-vertebrate host pairs. We are hopeful that these biotechnological products will make it to market in the near future.

## References

[1] Corral-Rodriguez M.A., Macedo-Ribeiro S., Barbosa Pereira P.J., Fuentes-Prior P. (2009). Tick-derived Kunitz-type inhibitors as antihemostatic factors. Insect Biochem. Mol. Biol..

[2] Chmelar J., Calvo E., Pedra J.H., Francischetti I.M., Kotsyfakis M. (2012). Tick salivary secretion as a source of antihemostatics. J. Proteom..

[3] Vector-Borne Diseases: Biology of Vector Host Relationship. https://grantome.com/grant/NIH/ZIA-AI000810-15.

[4] Karasuyama H., Miyake K., Yoshikawa S. (2020). Immunobiology of Acquired Resistance to Ticks. Front. Immunol..

[5] Jmel M.A., Aounallah H., Bensaoud C., Mekki I., Chmelar J., Faria F., M’Ghirbi Y., Kotsyfakis M. (2021). Insights into the Role of Tick Salivary Protease Inhibitors during Ectoparasite-Host Crosstalk. Int. J. Mol. Sci..

[6] Penalver E., Arillo A., Delclos X., Peris D., Grimaldi D.A., Anderson S.R., Nascimbene P.C., Perez-de la Fuente R. (2017). Parasitised feathered dinosaurs as revealed by Cretaceous amber assemblages. Nat. Commun..

[7] Sa-Nunes A., Oliveira C.J.F., Ribeiro J.M. (2020). Mast Cells and Basophils: From Malevolent Design to Coevolutionary Arms Race. Trends Parasitol..

[8] Simo L., Kazimirova M., Richardson J., Bonnet S.I. (2017). The Essential Role of Tick Salivary Glands and Saliva in Tick Feeding and Pathogen Transmission. Front. Cell. Infect. Microbiol..

[9] Nuttall P.A. (2019). Wonders of tick saliva. Ticks Tick-Borne Dis..

[10] Francischetti I.M., Sa-Nunes A., Mans B.J., Santos I.M., Ribeiro J.M. (2009). The role of saliva in tick feeding. Front. Biosci..

[11] Chmelar J., Kotal J., Langhansova H., Kotsyfakis M. (2017). Protease Inhibitors in Tick Saliva: The Role of Serpins and Cystatins in Tick-host-Pathogen Interaction. Front. Cell. Infect. Microbiol..

[12] Chmelar J., Kotal J., Karim S., Kopacek P., Francischetti I.M.B., Pedra J.H.F., Kotsyfakis M. (2016). Sialomes and Mialomes: A Systems-Biology View of Tick Tissues and Tick-Host Interactions. Trends Parasitol..

[13] Nuttall P.A., Labuda M. (2004). Tick-host interactions: Saliva-activated transmission. Parasitology.

[14] Oliva M.L., Sampaio M.U. (2009). Action of plant proteinase inhibitors on enzymes of physiopathological importance. An. Acad. Bras. Cienc..

[15] Stibraniova I., Bartikova P., Holikova V., Kazimirova M. (2019). Deciphering Biological Processes at the Tick-Host Interface Opens New Strategies for Treatment of Human Diseases. Front. Physiol..

[16] Page M.J., Di Cera E. (2008). Serine peptidases: Classification, structure and function. Cell. Mol. Life Sci..

[17] Ranasinghe S., McManus D.P. (2013). Structure and function of invertebrate Kunitz serine protease inhibitors. Dev. Comp. Immunol..

[18] Francischetti I.M., Valenzuela J.G., Andersen J.F., Mather T.N., Ribeiro J.M. (2002). Ixolaris, a novel recombinant tissue factor pathway inhibitor (TFPI) from the salivary gland of the tick, Ixodes scapularis: Identification of factor X and factor Xa as scaffolds for the inhibition of factor VIIa/tissue factor complex. Blood.

[19] Ribeiro J.M., Alarcon-Chaidez F., Francischetti I.M., Mans B.J., Mather T.N., Valenzuela J.G., Wikel S.K. (2006). An annotated catalog of salivary gland transcripts from Ixodes scapularis ticks. Insect Biochem. Mol. Biol..

[20] Kunitz M., Northrop J.H. (1936). Isolation from Beef Pancreas of Crystalline Trypsinogen, Trypsin, a Trypsin Inhibitor, and an Inhibitor-Trypsin Compound. J. Gen. Physiol..

[21] Ascenzi P., Bocedi A., Bolognesi M., Spallarossa A., Coletta M., De Cristofaro R., Menegatti E. (2003). The bovine basic pancreatic trypsin inhibitor (Kunitz inhibitor): A milestone protein. Curr. Protein. Pept. Sci..

[22] Mishra M. (2020). Evolutionary Aspects of the Structural Convergence and Functional Diversification of Kunitz-Domain Inhibitors. J. Mol. Evol..

[23] Flo M., Margenat M., Pellizza L., Grana M., Duran R., Baez A., Salceda E., Soto E., Alvarez B., Fernandez C. (2017). Functional diversity of secreted cestode Kunitz proteins: Inhibition of serine peptidases and blockade of cation channels. PLoS Pathog..

[24] Martins L.A., Kotal J., Bensaoud C., Chmelar J., Kotsyfakis M. (2020). Small protease inhibitors in tick saliva and salivary glands and their role in tick-host-pathogen interactions. Biochim. Biophys. Acta Proteins Proteom..

[25] de Magalhaes M.T.Q., Mambelli F.S., Santos B.P.O., Morais S.B., Oliveira S.C. (2018). Serine protease inhibitors containing a Kunitz domain: Their role in modulation of host inflammatory responses and parasite survival. Microbes Infect..

[26] Gomes M.T., Oliva M.L., Lopes M.T., Salas C.E. (2011). Plant proteinases and inhibitors: An overview of biological function and pharmacological activity. Curr. Protein Pept. Sci..

[27] Shamsi T.N., Parveen R., Fatima S. (2016). Characterization, biomedical and agricultural applications of protease inhibitors: A review. Int. J. Biol. Macromol..

[28] Ribeiro J.M.C., Mans B.J. (2020). TickSialoFam (TSFam): A Database That Helps to Classify Tick Salivary Proteins, a Review on Tick Salivary Protein Function and Evolution, With Considerations on the Tick Sialome Switching Phenomenon. Front. Cell. Infect. Microbiol..

[29] Costa G.C.A., Ribeiro I.C.T., Melo-Junior O., Gontijo N.F., Sant’Anna M.R.V., Pereira M.H., Pessoa G.C.D., Koerich L.B., Oliveira F., Valenzuela J.G. (2020). Amblyomma sculptum Salivary Protease Inhibitors as Potential Anti-Tick Vaccines. Front. Immunol..

[30] Reck J., Webster A., Dall’Agnol B., Pienaar R., de Castro M.H., Featherston J., Mans B.J. (2021). Transcriptomic Analysis of Salivary Glands of Ornithodoros brasiliensis Aragao, 1923, the Agent of a Neotropical Tick-Toxicosis Syndrome in Humans. Front. Physiol..

[31] Perez-Sanchez R., Carnero-Moran A., Soriano B., Llorens C., Oleaga A. (2021). RNA-seq analysis and gene expression dynamics in the salivary glands of the argasid tick Ornithodoros erraticus along the trophogonic cycle. Parasites Vectors.

[32] Oleaga A., Soriano B., Llorens C., Perez-Sanchez R. (2021). Sialotranscriptomics of the argasid tick Ornithodoros moubata along the trophogonic cycle. PLoS Negl. Trop. Dis..

[33] Garcia G.R., Gardinassi L.G., Ribeiro J.M., Anatriello E., Ferreira B.R., Moreira H.N., Mafra C., Martins M.M., Szabo M.P., de Miranda-Santos I.K. (2014). The sialotranscriptome of Amblyomma triste, Amblyomma parvum and Amblyomma cajennense ticks, uncovered by 454-based RNA-seq. Parasit Vectors.

[34] Karim S., Kumar D., Adamson S., Ennen J.R., Qualls C.P., Ribeiro J.M.C. (2021). The sialotranscriptome of the gopher-tortoise tick, Amblyomma tuberculatum. Ticks Tick-Borne Dis..

[35] Karim S., Ribeiro J.M. (2015). An Insight into the Sialome of the Lone Star Tick, Amblyomma americanum, with a Glimpse on Its Time Dependent Gene Expression. PLoS ONE.

[36] Maruyama S.R., Garcia G.R., Teixeira F.R., Brandao L.G., Anderson J.M., Ribeiro J.M.C., Valenzuela J.G., Horackova J., Verissimo C.J., Katiki L.M. (2017). Mining a differential sialotranscriptome of Rhipicephalus microplus guides antigen discovery to formulate a vaccine that reduces tick infestations. Parasite Vector.

[37] Araujo R.N., Silva N.C.S., Mendes-Sousa A., Paim R., Costa G.C.A., Dias L.R., Oliveira K., Sant’Anna M.R.V., Gontijo N.F., Pereira M.H. (2019). RNA-seq analysis of the salivary glands and midgut of the Argasid tick Ornithodoros rostratus. Sci. Rep..

[38] Chmelar J., Oliveira C.J., Rezacova P., Francischetti I.M., Kovarova Z., Pejler G., Kopacek P., Ribeiro J.M., Mares M., Kopecky J. (2011). A tick salivary protein targets cathepsin G and chymase and inhibits host inflammation and platelet aggregation. Blood.

[39] Branco V.G., Iqbal A., Alvarez-Flores M.P., Sciani J.M., de Andrade S.A., Iwai L.K., Serrano S.M., Chudzinski-Tavassi A.M. (2016). Amblyomin-X having a Kunitz-type homologous domain, is a noncompetitive inhibitor of FXa and induces anticoagulation in vitro and in vivo. Biochim Biophys Acta Proteins Proteom..

[40] Kolte D., Shariat-Madar Z. (2016). Plasma Kallikrein Inhibitors in Cardiovascular Disease: An Innovative Therapeutic Approach. Cardiol. Rev..

[41] Waxman L., Smith D.E., Arcuri K.E., Vlasuk G.P. (1990). Tick anticoagulant peptide (TAP) is a novel inhibitor of blood coagulation factor Xa. Science.

[42] Schaffer L.W., Davidson J.T., Vlasuk G.P., Siegl P.K. (1991). Antithrombotic efficacy of recombinant tick anticoagulant peptide. A potent inhibitor of coagulation factor Xa in a primate model of arterial thrombosis. Circulation.

[43] Karczewski J., Connolly T.M. (1996). The interaction of disagregin with the platelet fibrinogen receptor, glycoprotein IIb-IIIa. Blood.

[44] Decrem Y., Rath G., Blasioli V., Cauchie P., Robert S., Beaufays J., Frere J.M., Feron O., Dogne J.M., Dessy C. (2009). Ir-CPI, a coagulation contact phase inhibitor from the tick Ixodes ricinus, inhibits thrombus formation without impairing hemostasis. J. Exp. Med..

[45] Akagi E.M., de Sa Junior P.L., Simons S.M., Bellini M.H., Barreto S.A., Chudzinski-Tavassi A.M. (2019). Corrigendum to “Pro-apoptotic effects of Amblyomin-X in murine renal cell carcinoma “in vitro” [Biomed. Pharmacother. 66 (2012) 64-69]. Biomed. Pharmacother..

[46] Monteiro R.Q., Rezaie A.R., Bae J.S., Calvo E., Andersen J.F., Francischetti I.M. (2008). Ixolaris binding to factor X reveals a precursor state of factor Xa heparin-binding exosite. Protein Sci..

[47] Nazareth R.A., Tomaz L.S., Ortiz-Costa S., Atella G.C., Ribeiro J.M., Francischetti I.M., Monteiro R.Q. (2006). Antithrombotic properties of Ixolaris, a potent inhibitor of the extrinsic pathway of the coagulation cascade. Thromb. Haemost..

[48] Zhang H., Qiao R., Gong H., Cao J., Zhou Y., Zhou J. (2017). Identification and anticoagulant activity of a novel Kunitz-type protein HA11 from the salivary gland of the tick Hyalomma asiaticum. Exp. Appl. Acarol..

[49] Gao X., Shi L., Zhou Y., Cao J., Zhang H., Zhou J. (2011). Characterization of the anticoagulant protein Rhipilin-1 from the Rhipicephalus haemaphysaloides tick. J. Insect. Physiol..

[50] Cao J., Shi L., Zhou Y., Gao X., Zhang H., Gong H., Zhou J. (2013). Characterization of a new Kunitz-type serine protease inhibitor from the hard tick Rhipicephalus hemaphysaloides. Arch. Insect. Biochem. Physiol..

[51] Lai R., Takeuchi H., Jonczy J., Rees H.H., Turner P.C. (2004). A thrombin inhibitor from the ixodid tick, Amblyomma hebraeum. Gene.

[52] Blisnick A.A., Simo L., Grillon C., Fasani F., Brule S., Le Bonniec B., Prina E., Marsot M., Relmy A., Blaise-Boisseau S. (2019). The Immunomodulatory Effect of IrSPI, a Tick Salivary Gland Serine Protease Inhibitor Involved in Ixodes ricinus Tick Feeding. Vaccines.

[53] Almazan C., Fourniol L., Rakotobe S., Simo L., Borneres J., Cote M., Peltier S., Maye J., Versille N., Richardson J. (2020). Failed Disruption of Tick Feeding, Viability, and Molting after Immunization of Mice and Sheep with Recombinant Ixodes ricinus Salivary Proteins IrSPI and IrLip1. Vaccines.

[54] Carneiro-Lobo T.C., Konig S., Machado D.E., Nasciutti L.E., Forni M.F., Francischetti I.M., Sogayar M.C., Monteiro R.Q. (2009). Ixolaris, a tissue factor inhibitor, blocks primary tumor growth and angiogenesis in a glioblastoma model. J. Thromb. Haemost..

[55] Francischetti I.M., Mather T.N., Ribeiro J.M. (2004). Penthalaris, a novel recombinant five-Kunitz tissue factor pathway inhibitor (TFPI) from the salivary gland of the tick vector of Lyme disease, Ixodes scapularis. Thromb. Haemost..

[56] Valdes J.J., Schwarz A., Cabeza de Vaca I., Calvo E., Pedra J.H., Guallar V., Kotsyfakis M. (2013). Tryptogalinin is a tick Kunitz serine protease inhibitor with a unique intrinsic disorder. PLoS ONE.

[57] Paesen G.C., Siebold C., Dallas M.L., Peers C., Harlos K., Nuttall P.A., Nunn M.A., Stuart D.I., Esnouf R.M. (2009). An ion-channel modulator from the saliva of the brown ear tick has a highly modified Kunitz/BPTI structure. J. Mol. Biol..

[58] Soares T.S., Watanabe R.M., Tanaka-Azevedo A.M., Torquato R.J., Lu S., Figueiredo A.C., Pereira P.J., Tanaka A.S. (2012). Expression and functional characterization of boophilin, a thrombin inhibitor from Rhipicephalus (Boophilus) microplus midgut. Vet. Parasitol..

[59] Assumpcao T.C., Ma D., Mizurini D.M., Kini R.M., Ribeiro J.M., Kotsyfakis M., Monteiro R.Q., Francischetti I.M. (2016). In Vitro Mode of Action and Anti-thrombotic Activity of Boophilin, a Multifunctional Kunitz Protease Inhibitor from the Midgut of a Tick Vector of Babesiosis, Rhipicephalus microplus. PLoS Negl. Trop. Dis..

[60] Sasaki S.D., Azzolini S.S., Hirata I.Y., Andreotti R., Tanaka A.S. (2004). Boophilus microplus tick larvae, a rich source of Kunitz type serine proteinase inhibitors. Biochimie.

[61] Soares T.S., Oliveira F., Torquato R.J., Sasaki S.D., Araujo M.S., Paschoalin T., Tanaka A.S. (2016). BmTI-A, a Kunitz type inhibitor from Rhipicephalus microplus able to interfere in vessel formation. Vet. Parasitol..

[62] Florencio A.C., de Almeida R.S., Arantes-Costa F.M., Saraiva-Romanholo B.M., Duran A.F., Sasaki S.D., Martins M.A., Lopes F., Tiberio I., Leick E.A. (2019). Effects of the serine protease inhibitor rBmTI-A in an experimental mouse model of chronic allergic pulmonary inflammation. Sci. Rep..

[63] Lourenco J.D., Ito J.T., Cervilha D.A.B., Sales D.S., Riani A., Suehiro C.L., Genaro I.S., Duran A., Puzer L., Martins M.A. (2018). The tick-derived rBmTI-A protease inhibitor attenuates the histological and functional changes induced by cigarette smoke exposure. Histol Histopathol.

[64] Lourenco J.D., Neves L.P., Olivo C.R., Duran A., Almeida F.M., Arantes P.M., Prado C.M., Leick E.A., Tanaka A.S., Martins M.A. (2014). A treatment with a protease inhibitor recombinant from the cattle tick (Rhipicephalus Boophilus microplus) ameliorates emphysema in mice. PLoS ONE.

[65] Sasaki S.D., Tanaka A.S. (2008). rBmTI-6, a Kunitz-BPTI domain protease inhibitor from the tick Boophilus microplus, its cloning, expression and biochemical characterization. Vet. Parasitol..

[66] Duran A.F.A., Neves L.P., da Silva F.R.S., Machado G.C., Ferreira G.C., Lourenco J.D., Tanaka A.S., Martins M.A., Lopes F., Sasaki S.D. (2018). rBmTI-6 attenuates pathophysiological and inflammatory parameters of induced emphysema in mice. Int. J. Biol. Macromol..

[67] Islam M.K., Tsuji N., Miyoshi T., Alim M.A., Huang X., Hatta T., Fujisaki K. (2009). The Kunitz-like modulatory protein haemangin is vital for hard tick blood-feeding success. PLoS Pathog..

[68] Miyoshi T., Tsuji N., Islam M.K., Alim M.A., Hatta T., Yamaji K., Anisuzzaman Fujisaki K. (2010). A Kunitz-type proteinase inhibitor from the midgut of the ixodid tick, Haemaphysalis longicornis, and its endogenous target serine proteinase. Mol. Biochem. Parasitol..

[69] Alim M.A., Islam M.K., Anisuzzaman Miyoshi T., Hatta T., Yamaji K., Matsubayashi M., Fujisaki K., Tsuji N. (2012). A hemocyte-derived Kunitz-BPTI-type chymotrypsin inhibitor, HlChI, from the ixodid tick Haemaphysalis longicornis, plays regulatory functions in tick blood-feeding processes. Insect Biochem. Mol. Biol..

[70] Ceraul S.M., Dreher-Lesnick S.M., Mulenga A., Rahman M.S., Azad A.F. (2008). Functional characterization and novel rickettsiostatic effects of a Kunitz-type serine protease inhibitor from the tick Dermacentor variabilis. Infect. Immun..

[71] van de Locht A., Stubbs M.T., Bode W., Friedrich T., Bollschweiler C., Höffken W., Huber R. (1996). The ornithodorin-thrombin crystal structure, a key to the TAP enigma?. EMBO J..

[72] Karczewski J., Endris R., Connolly T.M. (1994). Disagregin Is a Fibrinogen Receptor Antagonist Lacking the Arg-Gly-Asp Sequence from the Tick, Ornithodoros-Moubata. J. Biol. Chem..

[73] Mans B.J., Louw A.I., Neitz A.W. (2002). Savignygrin, a platelet aggregation inhibitor from the soft tick Ornithodoros savignyi, presents the RGD integrin recognition motif on the Kunitz-BPTI fold. J. Biol. Chem..

[74] Ceraul S.M., Chung A., Sears K.T., Popov V.L., Beier-Sexton M., Rahman M.S., Azad A.F. (2011). A Kunitz protease inhibitor from Dermacentor variabilis, a vector for spotted fever group rickettsiae, limits Rickettsia montanensis invasion. Infect. Immun..

[75] Manen J.F., Simon P., Van Slooten J.C., Osteras M., Frutiger S., Hughes G.J. (1991). A nodulin specifically expressed in senescent nodules of winged bean is a protease inhibitor. Plant Cell.

[76] Levi M., van der Poll T., Buller H.R. (2004). Bidirectional relation between inflammation and coagulation. Circulation.

[77] Francischetti I.M., Seydel K.B., Monteiro R.Q. (2008). Blood coagulation, inflammation, and malaria. Microcirculation.

[78] Schechter M.E., Andrade B.B., He T., Richter G.H., Tosh K.W., Policicchio B.B., Singh A., Raehtz K.D., Sheikh V., Ma D. (2017). Inflammatory monocytes expressing tissue factor drive SIV and HIV coagulopathy. Sci. Transl. Med..

[79] Paesen G.C., Siebold C., Harlos K., Peacey M.F., Nuttall P.A., Stuart D.I. (2007). A tick protein with a modified Kunitz fold inhibits human tryptase. J. Mol. Biol..

[80] Hellman L., Akula S., Fu Z., Wernersson S. (2022). Mast Cell and Basophil Granule Proteases—In Vivo Targets and Function. Front. Immunol..

[81] Valdes J.J., Moal I.H. (2014). Prediction of Kunitz ion channel effectors and protease inhibitors from the Ixodes ricinus sialome. Ticks Tick-Borne Dis..

[82] Kettritz R. (2016). Neutral serine proteases of neutrophils. Immunol. Rev..

[83] Henriksen P.A. (2014). The potential of neutrophil elastase inhibitors as anti-inflammatory therapies. Curr. Opin. Hematol..

[84] Ferreira G.C., Bomediano Camillo L.M., Sasaki S.D. (2022). Structural and functional properties of rBmTI-A. A Kunitz-BPTI serine protease inhibitor with therapeutical potential. Biochimie.

[85] Batista I.F., Ramos O.H., Ventura J.S., Junqueira-de-Azevedo I.L., Ho P.L., Chudzinski-Tavassi A.M. (2010). A new Factor Xa inhibitor from Amblyomma cajennense with a unique domain composition. Arch. Biochem. Biophys..

[86] Pasqualoto K.F., Balan A., Barreto S.A., Simons S.M., Chudzinski-Tavassi A.M. (2014). Structural findings and molecular modeling approach of a TFPI-like inhibitor. Protein Pept. Lett..

[87] Drewes C.C., Dias R.Y., Hebeda C.B., Simons S.M., Barreto S.A., Ferreira J.M., Chudzinski-Tavassi A.M., Farsky S.H. (2012). Actions of the Kunitz-type serine protease inhibitor Amblyomin-X on VEGF-A-induced angiogenesis. Toxicon.

[88] Chudzinski-Tavassi A.M., De-Sa-Junior P.L., Simons S.M., Maria D.A., de Souza Ventura J., Batista I.F., Faria F., Duraes E., Reis E.M., Demasi M. (2010). A new tick Kunitz type inhibitor, Amblyomin-X, induces tumor cell death by modulating genes related to the cell cycle and targeting the ubiquitin-proteasome system. Toxicon.

[89] Ventura J.S., Faria F., Batista I.F., Simons S.M., Oliveira D.G., Morais K.L., Chudzinski-Tavassi A.M. (2013). A Kunitz-type FXa inhibitor affects tumor progression, hypercoagulable state and triggers apoptosis. Biomed. Pharmacother..

[90] Maria D.A., de Souza J.G., Morais K.L., Berra C.M., Zampolli Hde C., Demasi M., Simons S.M., de Freitas Saito R., Chammas R., Chudzinski-Tavassi A.M. (2013). A novel proteasome inhibitor acting in mitochondrial dysfunction, ER stress and ROS production. Invest. New Drugs.

[91] Ali A., Zeb I., Alouffi A., Zahid H., Almutairi M.M., Ayed Alshammari F., Alrouji M., Termignoni C., Vaz I.D.S., Tanaka T. (2022). Host Immune Responses to Salivary Components—A Critical Facet of Tick-Host Interactions. Front. Cell. Infect. Microbiol..

[92] Skare J.T., Garcia B.L. (2020). Complement Evasion by Lyme Disease Spirochetes. Trends Microbiol..

[93] Torina A., Villari S., Blanda V., Vullo S., La Manna M.P., Shekarkar Azgomi M., Di Liberto D., de la Fuente J., Sireci G. (2020). Innate Immune Response to Tick-Borne Pathogens: Cellular and Molecular Mechanisms Induced in the Hosts. Int. J. Mol. Sci..

[94] Willadsen P. (2004). Anti-tick vaccines. Parasitology.

[95] Andreotti R., Gomes A., Malavazi-Piza K.C., Sasaki S.D., Sampaio C.A., Tanaka A.S. (2002). BmTI antigens induce a bovine protective immune response against Boophilus microplus tick. Int. Immunopharmacol..

[96] Andreotti R. (2007). A synthetic bmti n-terminal fragment as antigen in bovine immunoprotection against the tick Boophilus microplus in a pen trial. Exp. Parasitol..

[97] Andreotti R., Cunha R.C., Soares M.A., Guerrero F.D., Leite F.P., de Leon A.A. (2012). Protective immunity against tick infestation in cattle vaccinated with recombinant trypsin inhibitor of Rhipicephalus microplus. Vaccine.

[98] de la Fuente J., Kocan K.M. (2022). The Impact of RNA Interference in Tick Research. Pathogens.

[99] Liao M., Zhou J., Gong H., Boldbaatar D., Shirafuji R., Battur B., Nishikawa Y., Fujisaki K. (2009). Hemalin, a thrombin inhibitor isolated from a midgut cDNA library from the hard tick Haemaphysalis longicornis. J. Insect. Physiol..

[100] Macedo-Ribeiro S., Almeida C., Calisto B.M., Friedrich T., Mentele R., Sturzebecher J., Fuentes-Prior P., Pereira P.J. (2008). Isolation, cloning and structural characterisation of boophilin, a multifunctional Kunitz-type proteinase inhibitor from the cattle tick. PLoS ONE.

[101] Valenzuela J.G., Francischetti I.M., Pham V.M., Garfield M.K., Mather T.N., Ribeiro J.M. (2002). Exploring the sialome of the tick Ixodes scapularis. J. Exp. Biol..

[102] Lobba A.R.M., Alvarez-Flores M.P., Fessel M.R., Buri M.V., Oliveira D.S., Gomes R.N., Cunegundes P.S., DeOcesano-Pereira C., Cinel V.D., Chudzinski-Tavassi A.M. (2022). A Kunitz-type inhibitor from tick salivary glands: A promising novel antitumor drug candidate. Front. Mol. Biosci..

